# Attention, predictions and expectations, and their violation: attentional control in the human brain

**DOI:** 10.3389/fnhum.2014.00490

**Published:** 2014-07-02

**Authors:** Simone Vossel, Joy J. Geng, Karl J. Friston

**Affiliations:** ^1^Wellcome Trust Centre for Neuroimaging, University College LondonLondon, UK; ^2^Cognitive Neuroscience, Institute of Neuroscience and Medicine (INM-3), Research Centre JülichJülich, Germany; ^3^Department of Psychology, Center for Mind and Brain, University of California DavisDavis, USA

**Keywords:** attentional networks, predictions, trial history, reward, emotions, neuroimaging, TMS, EEG

In the complex scenes of everyday life, our brains must select from among many competing inputs for perceptual synthesis—so that only the most relevant are fully processed and irrelevant (distracting) information is suppressed. At the same time, we must remain responsive to salient events outside our current focus of attention—and balancing these two processing modes is a fundamental task our brain constantly needs to solve.

This Research Topic examines how attentional control is guided by sensory predictions, prior knowledge, reward, task sets, and emotional factors. Moreover, the neural signatures of these mechanisms are investigated in Original Research Articles or summarized in Review, Perspective and Hypothesis and Theory Articles. Findings from a wide range of state-of-the-art complementary neuroscientific methods such as fMRI, M/EEG, TMS, and ALE-based meta-analysis are presented.

The collection of papers of this Research Topic provides an overview over our current knowledge in the field and also presents novel stimulating hypotheses on how attention is controlled in the human brain. It moreover bridges the gap to other disciplines such as decision-making and social and affective neuroscience.

## Conflict of interest statement

The authors declare that the research was conducted in the absence of any commercial or financial relationships that could be construed as a potential conflict of interest.

